# UVA, UVB Light Doses and Harvesting Time Differentially Tailor Glucosinolate and Phenolic Profiles in Broccoli Sprouts

**DOI:** 10.3390/molecules22071065

**Published:** 2017-06-26

**Authors:** Melissa Moreira-Rodríguez, Vimal Nair, Jorge Benavides, Luis Cisneros-Zevallos, Daniel A. Jacobo-Velázquez

**Affiliations:** 1 Tecnológico de Monterrey, Escuela de Ingeniería y Ciencias, Centro de Biotecnología FEMSA, Av. Eugenio Garza Sada 2501 Sur, C.P. 64849 Monterrey, NL, Mexico; melissamoreira19@hotmail.com (M.M.-R.); jorben@itesm.mx (J.B.); 2 Department of Horticultural Sciences, Texas A&M University, College Station, TX 77843-2133, USA; vimaln@tamu.edu (V.N.); lcisnero@tamu.edu (L.C.-Z.)

**Keywords:** UVA UVB light, UV radiation, abiotic stress, glucosinolate profiles, phenolic profiles, broccoli, sprouts

## Abstract

Broccoli sprouts contain health-promoting glucosinolate and phenolic compounds that can be enhanced by applying ultraviolet light (UV). Here, the effect of UVA or UVB radiation on glucosinolate and phenolic profiles was assessed in broccoli sprouts. Sprouts were exposed for 120 min to low intensity and high intensity UVA (UVA_L_, UVA_H_) or UVB (UVB_L_, UVB_H_) with UV intensity values of 3.16, 4.05, 2.28 and 3.34 W/m^2^, respectively. Harvest occurred 2 or 24 h post-treatment; and methanol/water or ethanol/water (70%, *v*/*v*) extracts were prepared. Seven glucosinolates and 22 phenolics were identified. Ethanol extracts showed higher levels of certain glucosinolates such as glucoraphanin, whereas methanol extracts showed slight higher levels of phenolics. The highest glucosinolate accumulation occurred 24 h after UVB_H_ treatment, increasing 4-methoxy-glucobrassicin, glucobrassicin and glucoraphanin by ~170, 78 and 73%, respectively. Furthermore, UVA_L_ radiation and harvest 2 h afterwards accumulated gallic acid hexoside I (~14%), 4-*O*-caffeoylquinic acid (~42%), gallic acid derivative (~48%) and 1-sinapoyl-2,2-diferulolyl-gentiobiose (~61%). Increases in sinapoyl malate (~12%), gallotannic acid (~48%) and 5-sinapoyl-quinic acid (~121%) were observed with UVB_H_ Results indicate that UV-irradiated broccoli sprouts could be exploited as a functional food for fresh consumption or as a source of bioactive phytochemicals with potential industrial applications.

## 1. Introduction

Broccoli sprouts constitute an exceptionally rich source of phenolic compounds and glucosinolates, with concentrations several times greater than those of mature counterparts [1,2]. Therefore, broccoli sprouts are considered a novel phytochemical-rich and plant-derived functional food [3].

Secondary metabolites are well known to be related to plant endogenous-defense mechanisms, being induced in response to biotic and abiotic stresses (e.g., wounding, ultraviolet (UV) light radiation and exogenous phytohormones), acting as natural phytoalexins to protect plants against these stresses [4,5]. Thus, plants can be used as biofactories of phytochemicals when applying a stress in order to accumulate high levels of secondary metabolites with potential industrial applications [6]. Abiotic stresses reported in broccoli and broccoli sprouts to induce the activation of phenolic and glucosinolate biosynthesis pathways include wounding [7], hypoxia and heat [8], UV light [9] and chemical treatments including methionine, tryptophan, salicylic acid [5], chitosan [5,10], methyl jasmonate [5,10,11], ethylene [11] and zinc sulphate [12].

Plants are unavoidably exposed to UV because they are sessile organisms and they need to capture sunlight for photosynthesis. It is well known that UV light causes different responses in plants, some of them are detrimental, including damage to DNA and proteins, generation of reactive oxygen species (ROS) and initiation of cellular stress responses, changes on cell physiology, as well as changes in plant growth, morphology and development [13]. Thus, they evolved mechanisms for UV protection and repair [14].

These mechanisms include deposition of UV-absorbing phenolic compounds in the outer epidermal layers and the production of antioxidant systems, action of reparative enzymes such as DNA photolyases, and expression of genes involved in both UV protection and repair [13]. UV regulates aspects of metabolism, modulates biochemical composition and thus, promotes the synthesis and accumulation of secondary metabolites, including phenolic compounds and glucosinolates [15]. Phenolics provide a UV-absorbing sunscreen that limits penetration of UVB into leaf tissues. On the other hand, glucosinolates, are not directly involved in UV protection. However, UV-mediated effects on glucosinolates are conceivable, since they are involved in the common plant defense response regulated by the signaling pathways involved in perception of UVB [9,16]. In this context, the application of UV stress has been reported in *Brassica* plants [9,17,18,19] as an approach to enhance the phytochemical content in vegetables to be used for fresh consumption or as functional and nutraceutical ingredients. However, research mainly focuses on the use of UVB radiation, on the mature vegetables, and on either glucosinolate or phenolics enhancement.

The demand of these compounds as nutraceuticals, food ingredients or topical ingredients, requires the use of extraction solvents carefully selected and handled to avoid toxicity for humans and danger the environment [20,21]. Thus, simultaneous extraction of phytochemicals (e.g., glucosinolates and phenolics) from broccoli sprouts using safer solvents would be attractive to several markets. Ethanol represents an advantageous option as extraction solvent, since botanical hydroalcoholic extracts used as active ingredients in the cosmetic and nutraceutical markets are typically ethanol-based given the toxicological constrains of other alcohols [22]. In this context, two hydroalcoholic solvents, one traditionally used to extract phytochemicals from broccoli (methanol/water; 70:30, *v*/*v*) [23] and one considered non-toxic for food and skin formulations (ethanol/water, 70:30, *v*/*v*) [24], were studied herein to extract both glucosinolate and phenolic compounds from broccoli sprouts exposed to UV.

The objectives of the present study were to determine how UVA and UVB light dose and harvest time after treatment could differentially enhance the accumulation of glucosinolate and phenolic compounds in 7-day-old broccoli sprouts and how feasible is an ethanolic extraction compared to methanol to obtain broccoli sprout extracts as attractive alternative for popular industrial markets including the fresh-food, cosmetic, skin care and dietary supplements markets.

## 2. Results

### 2.1. Effect of UVA and UVB Light on the Accumulation of Glucosinolates

**Figure 1:**
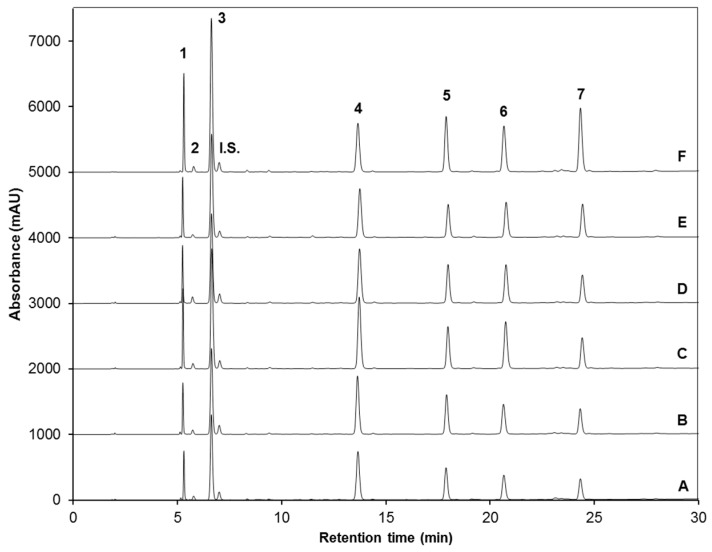
HPLC-DAD chromatograms (shown at 227 nm) of identified desulfoglucosinolates (dsg) from methanol/water (70:30, *v*/*v*) extracts of: (**A**) 7-day-old and (**B**) 8-day-old control broccoli sprouts, and 7-day-old broccoli sprouts treated with (**C**) UVA_L_, (**D**) UVA_H_, (**E**) UVB_L_ and (**F**) UVB_H_ and harvested 24 h after treatment. Peak assignment is shown in Table 1. Similar chromatographic profiles were obtained with ethanol/water (70:30, *v*/*v*) extracts. Glucoiberin-dsg (**1**); Progoitrin-dsg (**2**); Glucoraphanin-dsg (**3**); 4-hydroxy-glucobrassicin-dsg (**4**); Glucoerucin-dsg (**5**); Glucobrassicin-dsg (**6**); 4-methoxy-glucobrassicin-dsg (**7**); Internal standard, sinigrin (I.S.).

Young broccoli sprouts (7-day-old) were exposed to low and high intensity UVA and UVB lamps for 120 min of 3.16, 4.05, 2.28 and 3.34 W/m^2^ for treatments UVA_L_, UVA_H_, UVB_L_ and UVB_H_, respectively. Harvest took place 2 h and 24 h after treatment exposure. Upon methanolic or ethanolic extraction, desulfation and further chromatographic analysis, seven major glucosinolates were identified in both control and UV treated broccoli sprouts (Figure 1 and Table 1).

**Figure 2:**
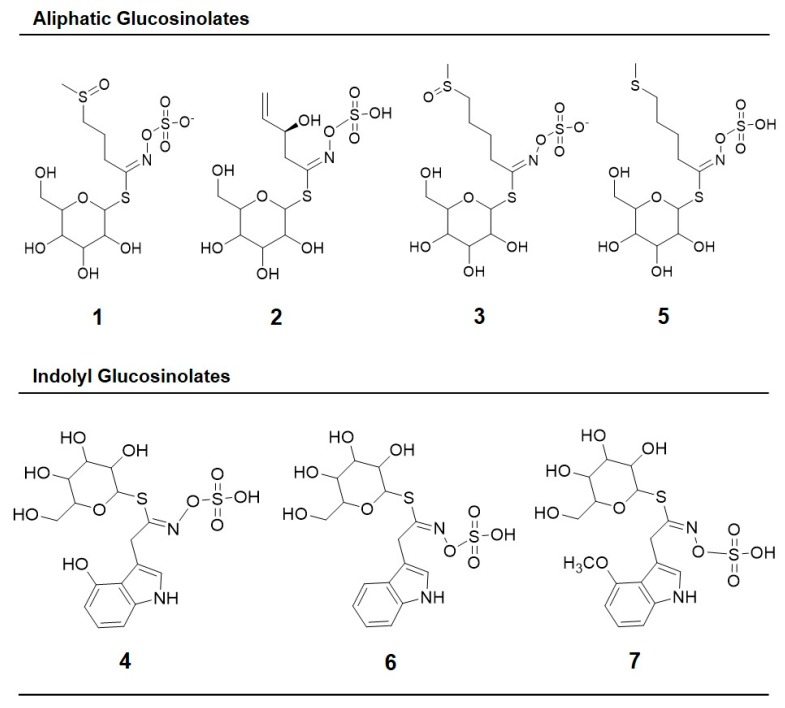
Chemical structures of glucosinolates identified after desulfation in broccoli sprouts subjected to UVA or UVB radiation stress: (**1**) Glucoiberin; (**2**) Progoitrin; (**3**) Glucoraphanin; (**4**) 4-hydroxy-glucobrassicin; (**5**) Glucoerucin; (**6**) Glucobrassicin; and (**7**) 4-methoxy-glucobrassicin. The numbering corresponds to the peak number assigned in Table 1.

The chemical structures of the identified glucosinolates after desulfation are shown in Figure 2 and include four aliphatic glucosinolates: glucoiberin (GIB, compound **1**); progoitrin (PRO, compound **2**); glucoraphanin (GRA, compound **3**); and glucoerucin (GER, compound **5**); and three indolyl glucosinolates: 4-hydroxy-glucobrassicin (4-HGBS, compound **4**); glucobrassicin (GBS, compound **6**); and 4-methoxy-glucobrassicin (4-MGBS, compound **7**). In general, glucoraphanin was the glucosinolate found in greater proportion in the sprouts, followed by 4-hydroxy-glucobrassicin (Figure 1).

The individual and total concentrations of glucosinolates (identified and quantified as desulfoglucosinolates) in 7-day-old broccoli sprouts subjected to UVA and UVB treatments are presented in Table 2. Overall, the methanolic or ethanolic extraction solvent did not affect the concentration of glucosinolates in broccoli sprouts. Nevertheless, in some treatments, extraction of certain individual glucosinolates were significantly (*p* < 0.05) enhanced with ethanol/water. For instance, higher concentration of GRA was observed when ethanol/water was used as extraction solvent (Table 2).

Treatment of 7-day-old broccoli sprouts with each UVA or UVB radiation doses induced the accumulation of glucosinolates in the seedlings; with exception for broccoli sprouts exposed to UVA_L_ and harvested 2 h after the treatment, in which concentration of glucosinolates remained unaltered compared with control sprouts (Table 2).

In general, the maximum UV-induced accumulation of glucosinolates was observed in broccoli sprouts harvested 24 h after the treatment, rather than 2 h (Table 2). The three main glucosinolates overproduced by UV stress were the aliphatic glucosinolates GIB and GRA, and the indolyl glucosinolates 4-HGBS and 4-MGBS (compounds **3**, **4** and **7** in Figure 1 and Figure 2).

Among all treatments, harvest of broccoli sprouts after 24 h of the application of UVB_H_ showed the highest enhancement (Figure 1F and Table 2), increasing the concentration of total glucosinolates by ~63% (108.79 ± 2.07 mmol/kg), compared to 8-day-old, control sprouts (66.56 ± 1.44 mmol/kg) with methanol as extraction solvent. Likewise, the concentration of the individual glucosinolates was greater in those sprouts treated with UVB_H_ light and harvested 24 h after the exposure. Compared to their respective 8-day-old control samples, the content of 4-MGBS, GIB, GBS, GRA, PRO, and GER increased by ~170, 89, 78, 73, 65 and ~39%, respectively. The content of 4-HGBS was not increased by this treatment (Table 2).

UVB_L_ (Figure 1E and Table 2) also induced accumulation of total and individual glucosinolates. However, contrary to the case of UVB_H_, results with UVB_L_ were more significant in samples harvested 2 h after the exposure to stress, rather than 24 h. Thereby, UVB_L_ with harvest 2 h afterwards increased total glucosinolate content by ~31% (81.75 ± 3.97 mmol/kg), compared to 7-day-old, control sprouts with methanol as extraction solvent. Likewise, under the same conditions, GBS, PRO, GIB, GRA, GER and 4-HGBS augmented by ~61, 47, 46, 33, 29 and 22%, respectively; while content of 4-MGBS remained unchanged.

Preceded by UVB_H_ treatment, UVA_L_ radiation 24 h after exposure (Figure 1C and Table 2) constitutes the second most promising treatment to enhance glucosinolate content, showing a ~36% increase of total glucosinolates (90.24 ± 3.38 mmol/kg) when compared to 8-day-old control sprouts extracted with methanol. The most affected glucosinolates in this case were PRO, GBS, GIB, GRA, 4-HGBS, 4-MGBS with increases of ~70, 59, 50, 44, 29 and 23%, respectively.

On the other hand, treatment with UVA_H_ light (Figure 1D and Table 2) showed higher concentration of glucosinolates with harvest of sprouts taking place 2 h after the UV treatment, similar to the case of UVB_L_ stress. Sprouts treated with UVA_H_ and harvested 2 h afterwards, showed a ~31% increase in total glucosinolate content (81.71 ± 1.38 mmol/kg), compared to 7-day-old control sprouts. Concentration of individual glucosinolates also showed similarities in trends noticed with UVB_L_ treatment, with the exceptions in PRO, which remained unaltered and 4-MGBS, which increased by ~20% after UVA_H_ treatment, compared to 7-day-old control sprouts.

### 2.2. Effect of UVA and UVB Light on the Accumulation of Phenolic Compounds

**Figure 3:**
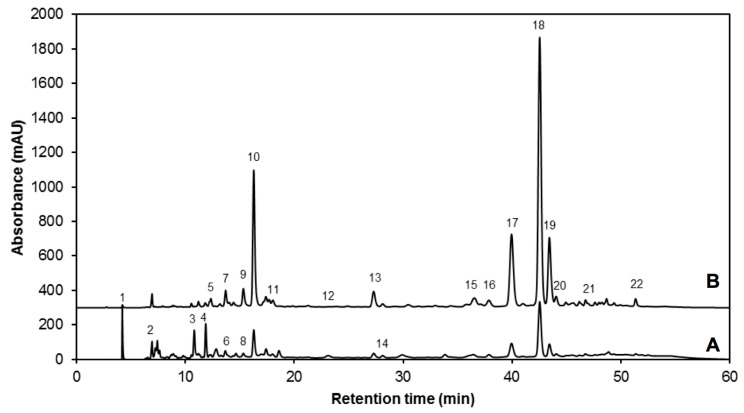
Typical HPLC-DAD chromatogram, shown at (**A**) 280 nm and (**B**) 320 nm of identified phenolic compounds from methanol/water (70:30, *v*/*v*) extracts of 7-day-old control broccoli sprouts. Peak assignment (as indicated in Table 3): Gallic acid hexoside I (**1**); gallotannic acid (**2**); *p*-hydroxybenzoic acid (**3**); gallic acid hexoside II (**4**); 4-*O*-caffeoylquinic acid (**5**); digalloyl hexoside (**6**); 3-*O*-hexoside kaempferol (**7**); gallic acid derivative (**8**); 1-*O*-sinapoyl-β-d-glucose (**9**); sinapoyl malate (**10**); 1,2-diferulolylgentiobiose (**11**); 5-sinapoylquinic acid (**12**); sinapic acid (**13**); gallic acid (**14**); kaempferol 3-*O*-sinapoyl-sophoroside 7-*O*-glucoside (**15**); 1,2-disinapoylgentiobiose (**16**); 1-sinapoyl-2′-ferulolylgentiobiose (**17**); 1,2,2′-trisinapoylgentiobiose (**18**); 1,2-disinapoyl-1′-ferulolylgentiobiose (**19**); 1,2-disinapoyl-2-ferulolylgentiobiose (**20**); 1-sinapoyl-2,2′-diferulolylgentiobiose (**21**); (isomeric) 1,2,2′-trisinapoylgentiobiose (**22**).

The phenolic content of the UV treated and control broccoli sprouts was also investigated. Twenty-two major phenolic compounds were identified in both control and UV treated broccoli sprouts (Figure 3 and Table 3).

**Figure 4:**
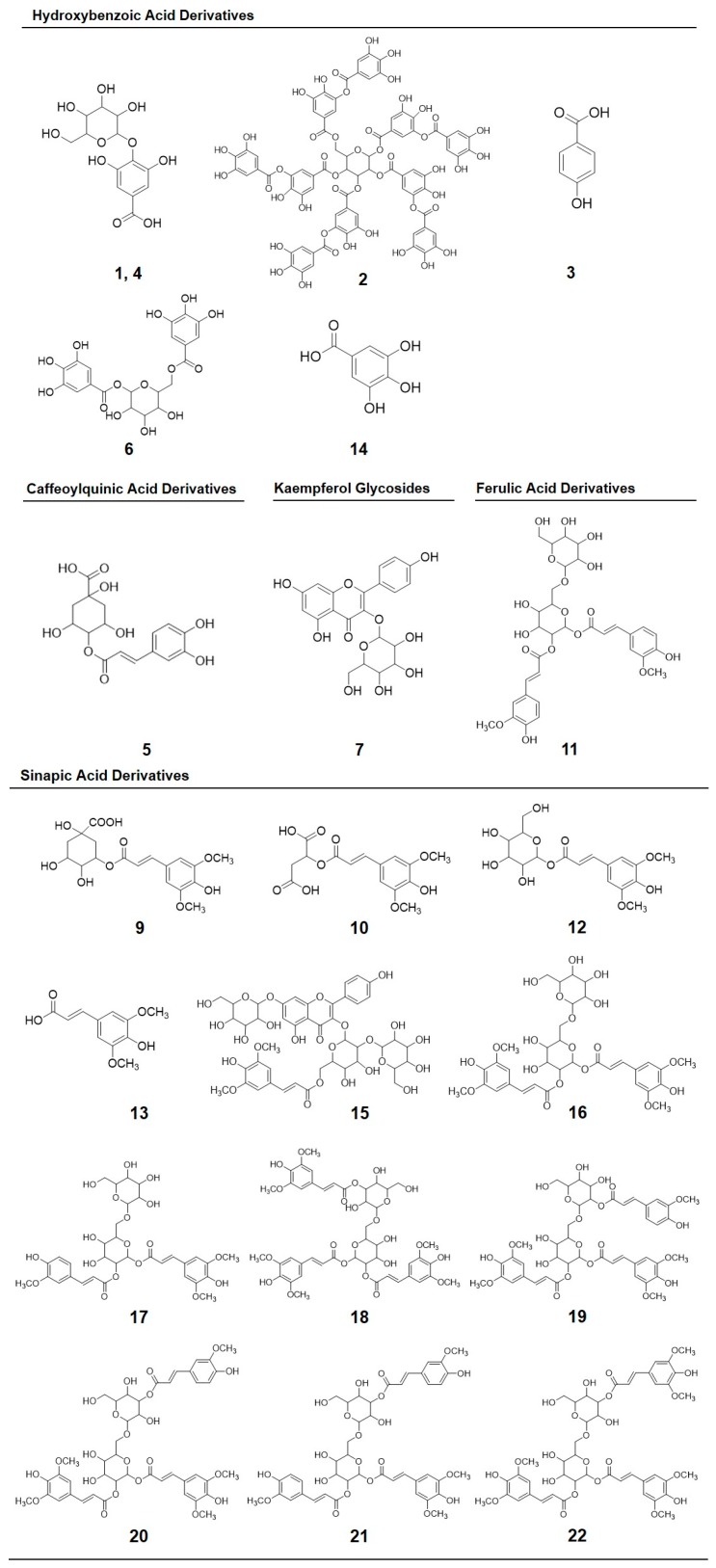
Chemical structures of phenolic compounds identified in broccoli sprouts subjected to UVA or UVB radiation stress: Gallic acid hexoside I (**1**); gallotannic acid (**2**); *p*-hydroxybenzoic acid (**3**); gallic acid hexoside II (**4**); 4-*O*-caffeoylquinic acid (**5**); digalloyl hexoside (**6**); 3-*O*-hexoside kaempferol (**7**); 1-*O*-sinapoyl-β-d-glucose (**9**); sinapoyl malate (**10**); 1,2-diferulolylgentiobiose (**11**); 5-sinapoylquinic acid (**12**); sinapic acid (**13**); gallic acid (**14**); kaempferol 3-*O*-sinapoyl-sophoroside 7-*O*-glucoside (**15**); 1,2-disinapoylgentiobiose (**16**); 1-sinapoyl-2’-ferulolylgentiobiose (**17**); 1,2,2’-trisinapoylgentiobiose (**18**); 1,2-disinapoyl-1’-ferulolylgentiobiose (**19**); 1,2-disinapoyl-2-ferulolylgentiobiose (**20**); 1-sinapoyl-2,2’-diferulolylgentiobiose (**21**); (isomeric) 1,2,2’-trisinapoylgentiobiose (**22**).The numbering corresponds to the peak number assigned in Table 3.

The chemical structures of the identified phenolics are shown in Figure 4, including gallic acid hexoside I (GAH I, 1) and gallic acid hexoside II (GAH II, **4**); gallotannic acid (GTA, compound **2**); *p*-hydroxybenzoic acid (*p*-HBA, **3**); 4-*O*-caffeoylquinic acid (4-*O*-CQA, **5**); digalloyl hexoside (diGH, **6**); 3-*O*-hexoside kaempferol (3-*O*-H-K, **7**); gallic acid derivative (GAD, **8**, not shown in Figure 4, derivative of compound **14**); 1-*O*-sinapoyl-β-d-glucose (1-*O*-S-β-d-g, **9**); sinapoyl malate (**10**); 1,2-diferulolylgentiobiose (1,2-diFG, **11**); 5-sinapoylquinic acid (5-SQA, **12**); sinapic acid (**13**); gallic acid (**14**); kaempferol 3-*O*-sinapoyl-sophoroside 7-*O*-glucoside (K-3-*O*-S-so-7-*O*-g, **15**); 1,2-disinapoylgentiobiose (1,2-diSG, **16**); 1-sinapoyl-2′-ferulolylgentiobiose (1-S-2-FG, **17**); 1,2,2′-tri-sinapoylgentiobiose and its isomer (1,2,2-triSG, **18**, **22**); 1,2-disinapoyl-1′-ferulolylgentiobiose (1,2-diS-1-FG, **19**); 1,2-disinapoyl-2-ferulolylgentiobiose (1,2-diS-2-FG, **20**); and 1-sinapoyl-2,2′-diferulolylgentiobiose (1-S-2,2-diFG, **21**). The individual and total concentrations of phenolic compounds in 7-day-old broccoli sprouts subjected to UVA and UVB treatments are presented in Table 4.

Unlike the pattern observed in the analysis of glucosinolates, with phenolics the solvent of extraction significantly affected (*p* < 0.05) the extraction of most compounds (except for 4-*O*-CQA and GAD), being methanol/water (70%, *v*/*v*) the most suitable solvent.

UV significantly (*p* < 0.05) induced the accumulation of ten of the twenty-two identified compounds (Table 4); particularly, UVA_L_ and UVB_H_ light were the main inducers. Compounds overproduced by UVA_L_ 2 h after UV treatment were GAH I, 4-*O*-CQA, GAD, sinapic acid and 1-S-2,2-diFG, with increases of ~14, 42, 48, 7 and 61%, as compared to 7-day-old control broccoli sprouts. UVA_L_ also enhanced the content of diGH, but only at 24 h after the stress treatment by ~22% compared to 8-day-old control sprouts (Table 4).

UVB also induced the synthesis of certain phenolic compounds (Table 4). UVB_H_ with harvest 24 h after the treatment caused increases in GTA (~48%), 5-SQA (~121%) and 1,2-diS-2-FG (~6%), compared to 8-day-old control broccoli sprouts.

UVB_L_ light with harvest of sprouts 2 h after the treatment increased the concentration of sinapoyl malate by ~12% compared to 7-day-old control sprouts. Additionally, UVB_L_ and harvest of the sprouts 2 h after treatment caused a significant (*p* < 0.05) ~20% increase of 1-S-2-FG when compared to its corresponding 7-day-old control (Table 4).

## 3. Discussion

### 3.1. Effect of Extraction Solvent on Phytochemical Profiles

The performance of methanol/water (70%, *v*/*v*) and ethanol/water (70%, *v*/*v*) as extraction solvents was tested in the present study. In general, the solvent did not affect the quantification of glucosinolates. Furthermore, it was demonstrated that both types of compounds, glucosinolates and phenolic compounds, were extracted simultaneously in an effective manner. This result could be related to the similar hydrophobicity of both hydroalcoholic solvents and the phytochemicals of interest. However, some exceptions were observed. For instance, the individual glucosinolate GRA showed slight higher (~13–20%) extraction yields when ethanol/water was used (Table 2). The latter is relevant since GRA is the major glucosinolate found in broccoli sprouts and typically, extraction of glucosinolates is performed using mixtures of methanol and water as extraction solvent [23]. Moreover, GRA is considered the most relevant aliphatic glucosinolate, as it is the precursor of anticancer isothiocyanate sulforaphane [25]. Thus, its extraction using ethanol/water (70%, *v*/*v*) could be of industrial interest.

Regarding the phenolic compounds, except for 4-*O*-CQA and GAD, the extraction was higher when methanol/water was used as extraction solvent (Table 4). The results observed agree with the effects of solvent on the extraction of polyphenols from broccoli, Brussels sprouts and white cabbage extracts, as methanol was found to be the most efficient solvent among 60% methanol, ethanol and acetone [20]. However, ethanol/water was the second most efficient solvent and, therefore, vegetable extracts (methanolic or ethanolic) were considered with potential to be utilized in food products with the aim of enhancing the quality and nutritive value of foods [20]. Moreover, ethanol represents an attractive option as extraction solvent in several markets (e.g., the cosmeceutical and nutraceutical markets), since botanical hydroalcoholic extracts used as active ingredients are typically ethanol-based given the possible toxicological reactions to methanol [22].

### 3.2. Effect of UVA and UVB Light on the Accumulation of Glucosinolates

Results show that supplementation of broccoli sprouts with UV increases the glucosinolate content. For instance, irradiation with high intensity UVB light (UVB_H_, 3.34 W/m^2^ for 120 min) in conjunction with harvest of sprouts 24 h after the treatment, showed the highest accumulation of both aliphatic and indolyl glucosinolates (Table 2). Similarly, in a previous report UVB light induced an accumulation of glucosinolates, mainly GRA and 4-MGBS in 12-day-old broccoli sprouts 24 h after exposure, triggered by an up-regulation in transcript levels of genes related to secondary metabolite biosynthesis pathways and stress response in the broccoli sprouts [9]. Other reports also agree that UVB light results in elevated glucosinolate levels in different plant species. For instance, in *Tropaeolum majus* L., UVB induced a 6-fold increase in the aromatic glucosinolate, glucotropaeolin [26]. Likewise, in *Arabidopsis thaliana* UVB elicited an increase of GRA and 4-MGBS [27]. Additionally, the present study showed that UVA light also increases the glucosinolate content in broccoli sprouts. Indeed, UVA_L_ (3.16 W/m^2^ for 120 min) treatment and harvest of sprouts 24 h afterwards, showed the second highest accumulation of both aliphatic and indolyl glucosinolates, with a pattern similar to UVB_H_ treatment (Table 2). The present results are novel, since information regarding UVA light on the accumulation of plant secondary metabolites is scarce, especially compared to existing literature on UVB induced compounds [28]. Moreover, the few reports on UVA radiation, have studied the effects mainly on phenolic compounds [29,30,31] with no reports on broccoli sprouts.

Regarding the effect of UVB light on the accumulation of glucosinolates, Mewis et al. [9] found that UVB increased the expression of genes coding for CYP71A and CYP71B families of Cyt P450 monooxygenases, involved in phytoalexin biosynthesis in *Arabidopsis thaliana.* Likewise, genes from the aliphatic glucosinolates biosynthetic pathway were also induced, the most responsive being *FMO GS-OX5*, involved in the oxidation of methylthioalkyl glucosinolates (e.g., GER, glucoibervirin) into methylsulfinylalkyl glucosinolates (e.g., GRA, GIB); and transcript levels of the transcription factor MYB51 were increased, as were gene transcripts involved in indolyl glucosinolates biosynthetic pathway, e.g., *CYP81F2*, which catalyzes the hydroxylation of GBS to 4-HGBS.

Therefore, the accumulation of GIB and GRA observed herein in both UVB and UVA treated sprouts might be due to the ability of UV to induce the expression of *FMO GS-OX5* gene. Moreover, the accumulation of 4-MGBS after UVB exposure in sprouts might be explained by the up-regulation of CYP81F2, the enzyme that converts GBS into 4-HGBS, the precursor of 4-MGBS. Interestingly, an acclimatization period of 2 h after UVB_L_ or UVB_H_ treatment was sufficient to induce the accumulation of GBS but not 4-MGBS; whereas only those sprouts harvested 24 h post-treatment showed increases in both GBS and especially 4-MGBS (Table 2). Regarding this finding, in the indolyl glucosinolate biosynthetic pathway, GBS is synthesized by sulfotransferases 16 and 18 (SOT16 and SOT18), and undergoes a conversion to 4-HGBS and later to 4-MGBS by the subfamily of *CYP81F* genes by hydroxylation and methylation reactions, respectively [32]. Thus, these observations suggest that in broccoli sprouts the 4-hydroxylation and further methylation of GBS are favored by UVB light (rather than UVA light) and longer periods of acclimatization (24 h rather than 2 h), as observed by an accumulation of 4-MGBS.

It is also known that the plant responses to UVB partially overlap those of defense signaling induced by insects and pathogens [33]. Particularly in broccoli sprouts, UVB radiation induces the up-regulation of pathogen- and salicylic acid (SA)-responsive genes *PR-1* and *PR-*2, in addition to genes associated with salicylate and jasmonic acid (JA) signaling, with pathogen attack and/or wounding, such as *PR-4* and *BG3*, leading to the production of glucosinolates as a plant defense mechanism [9].

On the other hand, an UVB-specific signaling pathway associated with photoreceptors is known to be activated in plants under UVB conditions. In this pathway, UV RESISTANCE LOCUS 8 (UVR8) interacts with the E3 ubiquitin ligase CONSTITUTIVELY PHOTOMORPHOGENETIC 1 (COP1) to induce the transcription factor ELONGATED HYPOCOTYL 5 (HY5), which in turn regulates genes involved in photomorphogenetic UVB response and metabolite accumulation [16].

Altogether, it is likely that in the present study, UVB_H_ triggers the expression of the broccoli sprouts’ secondary metabolism via the simultaneous activation of the UVR8-COP1-HY5 and the JA-/SA-/ROS-related signaling pathways. Regarding UVA light, it is likely that at lower doses, a similar mechanism culminates in the induction of genes related to both aliphatic and indolyl biosynthetic pathways, perhaps with the signal transduction cascade being led by the UVA-specific photoreceptors, such as cryptochromes (CRY) and phototropins [28].

Moreover, Kusano et al. [34] reported that glucosinolates accumulate relatively late during an “acclimatization process”, rather than being an immediate response. UVB exposure triggers substantial reprogramming of primary metabolism, accumulating “rapid response” primary metabolites which, in turn, prime the cell to facilitate the later production of secondary metabolites [16,34]. Thus, partially explaining the fact that the greatest accumulation of glucosinolates was observed 24 h and not 2 h after the UVB_H_ treatment.

Interestingly, UVB_L_ (2.28 W/m^2^ for 120 min) and UVA_H_ (4.05 W/m^2^ for 120 min) treatments also caused the accumulation of glucosinolates, exhibiting similar effects at 2 h and 24 h after the treatments. In both cases, however, the accumulation of glucosinolates was significantly higher when sprouts were harvested 2 h after UV application. The latter could be explained from the perspective of kinetics where for UVB_H_ the kinetics of glucosinolate biosynthesis is higher than use throughout the period of acclimatization evaluated, whereas for UVB_L_ (and UVA_H_) the kinetics of biosynthesis is high only at the beginning of the acclimatization process (2 h) but then the kinetics of use overcomes at late time (24 h). A similar effect was observed in the work of Mewis et al. [9], in which the lower the UVB dose, the lower the glucosinolate content quantified 24 h post-treatment compared to 2 h in 12-day-old broccoli sprouts. Finally, for UVA_L_ the kinetics of biosynthesis overcomes the use only at late time, thus accumulating glucosinolates at 24 h post-treatment.

**Figure 5:**
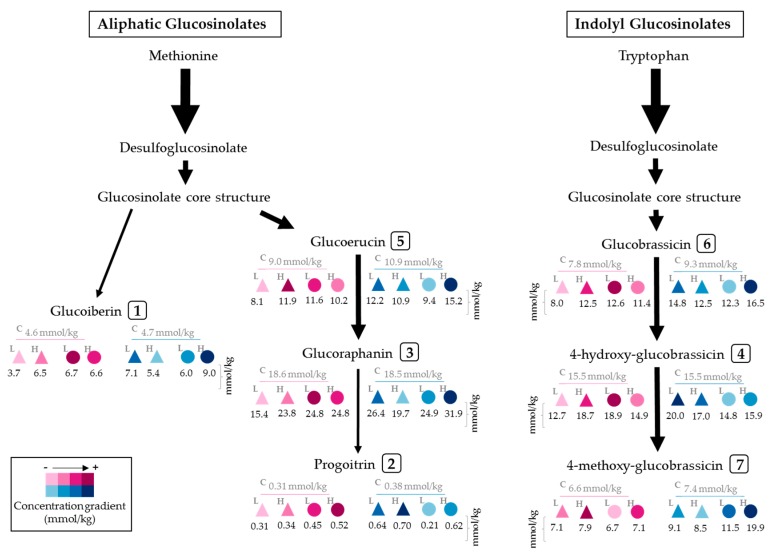
Accumulation of individual glucosinolates in broccoli sprouts treated with UV light. Identified compounds are located in the glucosinolate biosynthetic pathway. The numbering of compounds corresponds to the peak number assigned in Table 1. UV treatments are represented as follows: the type of light applied was UVA (triangle) or UVB (circle); UV dose was low (L) (3.16 and 2.28 W/m^2^ for 120 min for UVA_L_ and UVB_L_, respectively), high (H) (4.05 and 3.34 W/m^2^ for 120 min for UVA_H_ and UVB_H_, respectively), or 0 W/m^2^ for controls (C). Harvest of sprouts took place 2 h (pink) or 24 h (blue) after the UV treatment. The darker the color, the greater the compound’s accumulation after a given treatment. Concentrations (in mmol/kg) correspond to data from methanolic extracts presented in Table 2.

A schematic representation of the individual glucosinolates accumulated in broccoli sprouts treated with UV light is shown in Figure 5. The arrows in the diagram emphasize the direction of the carbon flux through the biosynthetic pathway. Additionally, it serves as a visual tool to select a treatment to enhance the content of a desired glucosinolate. For instance, the deeper shade of blue below compounds GIB, GER, GRA, GBS and 4-MGBS leads to the conclusion that application of UVB_H_ light + harvest 24 h post-treatment may be used to accumulate such phytochemicals in broccoli sprouts (Figure 5).

The accumulated aliphatic and indolyl glucosinolates in UVB and UVA stressed broccoli sprouts have a broad range of industrial applications. For instance, in the fresh-food industry, the pharmaceutical and the dietary supplements industries, glucoraphanin has gained attention in the last years due to the anticarcinogenic properties of its breakdown product, sulforaphane [5,25]. In addition, glucosinolates from UV treated broccoli sprouts can also be utilized by the cosmetic industry as natural active ingredients for skin photoprotection [35] and by the agricultural industry as natural insecticides to protect horticultural crops from pathogen attacks [17].

### 3.3. Effect of UVA and UVB Light on the Accumulation of Phenolic Compounds

The phenolic profile of broccoli sprouts obtained herein agrees with previous reports performed with broccoli inflorescences, broccoli sprouts, as well as other related *Brassica olereacea* vegetables [7,11,20,25,36,37,38,39,40]. In the present study, the main phenolic compounds found in broccoli sprouts are flavonol glycosides, and hydroxycinnamic acids (e.g., ferulic acid, sinapic acid), hydroxybenzoic acids (e.g., gallic acid, *p*-hydroxybenzoic acid), derivatives of these phenolic acids, and some hydrolysable tannins. Other authors also report the presence of protocatechuic acid, *p*-coumaric acid and specific flavonols (quercetin and kaempferol) as major phenolic compounds in broccoli and broccoli sprouts [3,9,40]. However, these were not detected in the present work, except for a couple kaempferol glycosides (Figure 4). Differences between the phenolic profiles obtained herein and those previously reported could be attributed to multiple factors, including genetic variances between cultivars, maturity of the vegetable, growing conditions, and even the methods of analysis and extraction parameters (e.g., solvent applied) [36,41].

It is known that UVB induces *CHS* and other genes involved in the phenylpropanoid biosynthesis pathway [42]. UV light absorbing flavonoids, hydroxycinnamic acids and other phenolic compounds are produced and further incorporated in the epidermis, where they play an important role in plant tolerance to UVB due to their ability to reduce UV penetration into the plant tissue (UV screening properties), to act as antioxidants to protect the plant from damage caused by UV-induced ROS [13,14,31] and to be involved in defense against herbivorous insects and pathogens [43].

Therefore, an increase in phenolic compounds after UVB exposure was expected in the present study. In fact, UVB_L_ treated sprouts harvested 2 h after treatment showed a significant (*p* < 0.05) increase of ~6% in total phenolic content compared to control sprouts harvested at the same time (Table 4). Such increase reflects the accumulation of individual phenolics, mainly the sinapic acid derivatives 1-S-2-FG (~20%) and sinapoyl malate (~12%). The first compound has been reported to increase when exogenous ethylene is applied to wounded broccoli florets [11] and it was attributed to an ethylene-induced expression of genes related with phenolics and lignin biosynthesis in wound-stressed plants [11,44]. Furthermore, it has been stated that UVB induces the production of stress signaling molecules, such as endogenous ethylene [42]. Therefore, it is likely that broccoli sprouts irradiated with UVB respond by producing ethylene, which acts as a signaling molecule to upregulate stress-related genes and phenylpropanoid-related genes, and ultimately triggering the accumulation of UV absorbing phenolics such as 1-S-2-FG, which was enhanced by both doses of UVB tested herein.

Moreover, sinapate esters or sinapates (e.g., sinapoyl malate) are considered among the primary class of molecules screening UVB in the leaf epidermis of plants, especially *Brassicaceae* plants [45]. In the present study, the increase (~12%) in sinapoyl malate content in UVB treated sprouts agrees with previous reports performed in *Arabidopsis thaliana* and *Brassica napus*, where UVB radiation induced the accumulation of sinapoyl malate and other sinapates that strongly absorb in the UV range, and thus protect the plant against environmentally relevant UVB radiation [46]. The above is particularly relevant for the cosmetic industry since sinapoyl malate has recently gained recognition as a potential plant-derived UVB sunscreen molecule to be incorporated into sun-protection products [45,47]. Interestingly, UVA_H_ also induced a ~7–18% increase in sinapoyl malate concentration, while UVA_L_ triggered an accumulation of its precursor, sinapic acid, as an early response. Thus, it is possible that sinapate esters also play an important UV absorbing role against UVA radiation in young broccoli sprouts.

UVB_H_ with harvest 24 h post-treatment also induced the synthesis of other sinapic acid derivatives in the sprouts, particularly, 5-SQA (~121%) and 1,2-diS-2-FG (~6%), as compared to 8-day-old control sprouts, respectively. As proposed for 1-S-2-FG, the accumulation of these hydroxycinnamic acid derivatives might be a consequence of the UVB-stimulated production of, not only ethylene, but also hydrogen peroxide (H_2_O_2_), which also acts as a signaling molecule in the transduction of UV-induced stress signals to activate downstream target genes encoding for peroxidases and genes related to the biosynthesis of phenolics, among others [48]. Additionally, the fact that this treatment increased the content of GTA (~48%) was partially expected, since GTA derives from gallic acid, an hydroxybenzoic acid that absorbs UV light in the range of UVB (~275–280 nm) [49].

The only chlorogenic acid (CGA) derivative identified herein, 4-*O*-CQA, was accumulated by ~42% in sprouts treated with UVB_L_, harvested 24 h afterwards and using methanol as extraction solvent; and by ~127% in sprouts treated with UVA_L_, harvested 2 h after and extracted with ethanol. The above agrees with previous reports where Tegelberg et al. [50] demonstrated an increase in caffeoylquinic acid in silver birch (*Betula pendula*) seedlings exposed to slightly above-ambient UVB radiation. Likewise, in tomato (*Solanum lycopersicum*), UVB exposure induced an increase in CGA and several of its isomers, correlating with an overexpression of hydroxycinnamoyl CoA quinate transferase (HQT), the key enzyme catalyzing the biosynthesis of CGAs [51].

Moreover, higher levels of lignin precursors, including 4-*O*-CQA and glycosides of hydroxycinnamic acids (e.g., 1,2-diFG, 1,2-diS-2-FG, 1,2,2-triSG), in broccoli florets and potatoes subjected to abiotic stresses have been associated with the stress-induced activation of the phenylpropanoid metabolism required for the biosynthesis of lignin that serves as a water impermeable barrier preventing excessive water loss [11,52]. This, partially explains the increases observed in these phenolics after UV treatments.

As stated for glucosinolates, the accumulation of phenolic compounds in UVB treated broccoli sprouts may be also attributed to the activation of the UVR8-COP1-HY5 signaling pathway [13], which has been proven to trigger the overexpression of genes coding for key enzymes of the phenolic biosynthetic pathway, including phenylalanine ammonia-lyase (PAL) and various flavonol synthases, as has been previously reported in *A. thaliana* [53] after UVB exposure.

Regarding the kaempferol glycosides K-3-*O*-S-so-7-*O*-g and 3-*O*-H-K, the slight increase in their concentration (*p* < 0.05, compared to 7-day-old controls) after UVB exposure can be related to an up-regulation of genes homologous to the UDP-glycosyltransferase family protein, *UGT73B2*, which catalyzes the glycosylation of flavonoids from UDP-glucose; based on Mewis et al. [9] who reported a 3.5-fold gene induction 24 h after the UVB treatment of 12-day-old broccoli sprouts. Moreover, UVB could also be stimulating the production of nitric oxide (NO), which may reduce the levels of UVB-induced ROS and up-regulate the expression of HY5 and its final target genes such as *CHS* [54] hence, accumulating flavonoids and derivatives to absorb UVB and also to scavenge ROS, as reported in maize sprouts [55]. Once again, these responses were also observed in sprouts treated with UVA radiation, supporting the idea that these mechanisms may not be exclusive to UVB radiation.

Contrary to UVB, less is known about the effects of UVA light, however, a few reports have demonstrated that UVA radiation can induce the accumulation of phenolic compounds in plants such as *Rosa hybrida* and *Fuschia hybrida* [29], *Phaseoulus mungo* [30], *Betula pendula* [31] and *Daucus carota* [56]. Therefore, an increase in the phenolic content of UVA irradiated broccoli sprouts was also expected herein. Interestingly, most of the compounds increased by UVB_H_ treatment were also increased by UVA_L_, especially at 2 h after UV treatment (Table 4). However, most of the hydroxybenzoic acid derivatives detected were enhanced only by this radiation, i.e. GAH I, GAD and diGH, being increased by ~14, 48 and 33% as compared to 7-day-old control broccoli sprouts. Given their maximum wavelength of absorption at 280 nm, these compounds were not expected to be primarily increased by UVA radiation (320–400 nm); however, it has been previously reported that UVA induces the accumulation of gallic acid derivatives, such as theogallin [57].

Regarding the mechanisms governing the UVA induced accumulation of phenolic compounds, in a similar manner than UVB, it is likely that they involve: UVA induced transcript accumulation of genes involved in the phenylpropanoid pathway; UVA induced activation of PAL; and UVA induced accumulation of phenolic compounds via specific photoreceptors such as CRY [28]. Additionally, given the similar effects herein observed between UVB and UVA treatments, it cannot be ruled out the possible interactions between UVB specific UVR8 receptor and UVA signaling pathways controlling metabolite accumulation in plants, plus other mechanisms so far only elucidated for UVB radiation, including the role of NO and ethylene.

**Figure 6:**
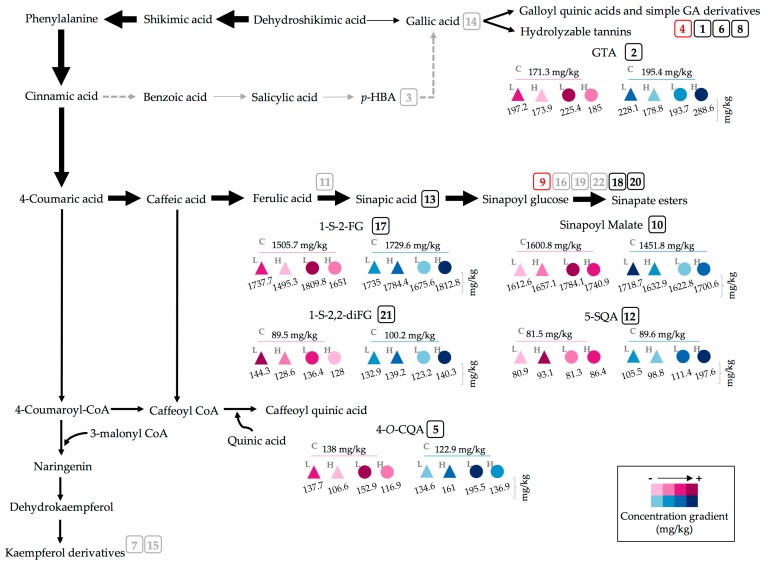
Accumulation of individual phenolic compounds in broccoli sprouts treated with UV light. Identified compounds are located in the phenolic biosynthetic pathway. Dashed arrows represent multiple enzymatic steps. The numbering of compounds corresponds to the peak number assigned in Table 3. Numbers in red correspond to compounds whose phenolic concentration decreased by all treatments; in gray, remained unaffected; and in black, increased. From the latter group, compounds 2, 5, 10, 12, 17 and 21 were taken as the most representatives. UV treatments are represented as follows: the type of light applied was UVA (triangle) or UVB (circle); UV dose was low (L) (3.16 and 2.28 W/m^2^ for 120 min for UVA_L_ and UVB_L_, respectively), high (H) (4.05 and 3.34 W/m^2^ for 120 min for UVA_H_ and UVB_H_, respectively), or 0 W/m^2^ for controls (C). Harvest of sprouts took place 2 h (pink) or 24 h (blue) after the UV treatment. The darker the color, the greater the compound’s accumulation after a given treatment. Concentrations (in mg/kg) correspond to data from methanolic extracts presented in Table 4. Abbreviations: Gallic acid (GA), gallotannic acid (GTA), *p*-hydroxybenzoic acid (*p*-HBA), 4-*O*-caffeoylquinic acid (4-*O*-CQA), 5-sinapoylquinic acid (5-SQA), 1-sinapoyl-2′-ferulolylgentiobiose (1-S-2-FG), 1-sinapoyl-2,2′-diferulolylgentiobiose (1-S-2,2-diFG).

A schematic representation of the individual phenolic compounds accumulated in broccoli sprouts treated with UV light is shown in Figure 6. The arrows in the diagram emphasize the direction of the carbon flux through the biosynthetic pathway. As a visual tool to select one or more treatments to enhance the content of desired phenolics, this diagram facilitates identification of treatment with UVB_L_ + harvest 24 h post-treatment to accumulate 4-*O*-CQA or UVA_L_ + harvest 24 h afterwards to accumulate sinapoyl malate in broccoli sprouts (Figure 6).

Given the increasing data supporting the role of phenolics in preserving human health, the production of phenolic compounds in broccoli sprouts would be of great interest for the cosmetic, pharmaceutical and food industry. For instance, sinapoyl malate has been recognized as a natural sunscreen agent [47], 4-*O*-CQA has been associated with the reduction of the risk of developing chronic diseases such as type II diabetes, cardiovascular and neurodegenerative diseases [58]. Likewise, sinapic acid ferulic acid and the phenolic aglycones of 1,2-diSG, 1-S-2-FG, 1,2,2-triSG, 1,2-diFG, and 1,2-diS-2-FG, are important antioxidants that inhibit the peroxidation of low density lipoproteins, preventing the progression of atherosclerosis [59].

## 4. Materials and Methods

### 4.1. Chemical and Plant Material

Sulfatase (from *Helix pomatia*), sinigrin hydrate, sephadex A-25, sodium acetate, orthophosphoric acid, sinapic acid, ferulic acid, and gallic acid and 3-*O*-caffeoylquinic acid (3-*O*-CQA) were obtained from Sigma-Aldrich Co. (St. Louis, MO, USA) and desulfoglucoraphanin was obtained from Santa Cruz Biotechnology (Dallas, TX, USA). Acetonitrile (HPLC grade) and methanol (HPLC grade) were obtained from Desarrollo de Especialidades Químicas, S.A. de C.V (Monterrey, NL, México), and ethanol (HPLC grade) was from Control Técnico y Representaciones, S.A. de C.V (Monterrey, México). Deionized water (18.2 MΩ·cm resistance) was used in all procedures and was obtained from a Milli-Q Element water purification system (Millipore, Bedford, MA, USA).

Broccoli (*Brassica oleracea* L., var. *italica*, cv. Waltham 29) seeds, Sun Gro Horticulture’s Canadian *Sphagnum* peat moss substrate and Landmark Plastic Corporation’s propagation trays were obtained from IMAISA (Monterrey, NL, México).

### 4.2. Sprouting Method and UV Treatments

The sprouting method was adapted from Martínez-Villaluenga et al. [1]. Briefly, broccoli seeds (0.5 g per replication) were sanitized for 15 min in sodium hypochlorite (1.5%, *v*/*v*), rinsed with Milli-Q water and soaked with aeration overnight in darkness and at room temperature. After pouring off the soaking water, the seeds were spread evenly on standard 200 square cell plug trays (21.38″ × 11.05″ × 1.75″) containing Canadian Sphagnum peat moss previously moistened. Sprouts were grown in a culture room with controlled temperature (25 °C) and a photoperiod regime with cycles of 16 h light and 8 h darkness. Water was atomized every 12 h until the 7th day after sowing.

UV treatments set-up was based on Mewis et al. [9] with slight adjustments. Four UV treatments were carried out in special UVA and UVB chambers with 7-day-old sprouts. Chambers were equipped with a single 20 W (for low intensity) or 40 W (for high intensity) UVA or UVB lamp. Low intensity UVA (UVA_L_) lamp was a Sylvania F20W T12 BL350 (Ledvance LLC., Wilmington, MA, USA); high intensity UVA (UVA_H_) lamp, a Sylvania F40W T12 BL350 (Ledvance LLC); low intensity UVB (UVB_L_) lamp, a Philips TL 20W/12 RS (Philips, Ljubljana, Slovenia); and high intensity UVB (UVB_H_) lamp, a Philips TL 40 W/12 RS (Philips). Trays with broccoli sprouts were placed 30 cm below the irradiation source. All treatments consisted of a single UV exposure for 120 min, of 3.16, 4.05, 2.28 and 3.34 W/m^2^ for treatments UVA_L_, UVA_H_, UVB_L_ and UVB_H_, respectively. The irradiation was determined prior to the experiment with a PMA 2200 radiometer equipped with PMA 2110 UVA and PMA 2106 UVB sensors (Solar Light, Glenside, PA, USA) measuring in the spectral range from 320–400 nm and 280–320 nm, respectively. After UV treatments, trays were returned to culture room. Sprouts were harvested 2 or 24 h after treatment application, immediately flash-frozen in liquid nitrogen, placed at −80 °C until freeze-dried (Labconco, Kansas City, MO, USA), and then ground to a fine powder. Samples were stored at −80 °C until further analysis.

### 4.3. Phytochemical Analyses

#### 4.3.1. Extraction of Phytochemicals

A single procedure was performed to extract both the glucosinolates and the phenolic compounds from the freeze-dried broccoli sprouts. To evaluate the effect of solvent composition over simultaneous glucosinolate and phenolic compounds extraction yield, two different hydroalcoholic mixtures were studied including a methanol/water (70:30, *v*/*v*) and an ethanol/water (70:30, *v*/*v*) extraction.

The extraction of phytochemicals and further desulfation of glucosinolates, was performed as described by Villarreal-García et al. [11]. Briefly, 10 mL of methanol/water (70:30, *v*/*v*) or ethanol/water (70:30, *v*/*v*) previously heated for 10 min at 70 °C in a reciprocating water bath (VWR, Radnor, PA, USA), were added to broccoli sprouts powder (0.2 g) followed by the addition of 50 µL of a 3 mM solution of sinigrin as internal standard (I.S). To ensure myrosinase inactivation, samples were incubated at 70 °C for 30 min and vortexed at 0, 10 and 20 min. The extracts were removed from the water bath, left to cool at room temperature and centrifuged (18,000× *g*, 10 min, 4 °C). The clarified extract (supernatant) was recovered for glucosinolates and phenolic compounds analysis.

#### 4.3.2. Analysis of Glucosinolates

##### Desulfation of Glucosinolates

Broccoli sprouts glucosinolates were analyzed using a method that converts the glucosinolates to the equivalent desulfoglucosinolates. Therefore, immediately after the extraction of phytochemicals, glucosinolates were desulfated and purified using disposable polypropylene columns (Thermo Fisher Scientific, Waltham, MA, USA). Columns were prepared by adding 0.5 mL of water, followed by 0.5 mL of previously prepared Sephadex A-25 and an additional 0.5 mL of water. Clarified methanolic or ethanolic extract supernatant (3 mL) were added into a prepared column and allowed to drip through slowly. Columns were washed with 2 × 0.5 mL of water followed by 2 × 0.5 mL of 0.02 M sodium acetate. Purified sulfatase (75 μL) was added to each sample and left at room temperature overnight (12 h). Desulfoglucosinolates were eluted with a total of 1.25 mL of water (0.5 mL + 0.5 mL + 0.25 mL).

##### Identification and Quantification of Desulfoglucosinolates by High-Performance Liquid Chromatography-Diode Array Detection (HPLC-DAD) and HPLC-Electrospray Ionization (ESI)-MS^n^

Determination of desulfoglucosinolates was performed as reported by Vallejo et al. [25] with slight modifications described by Villarreal-García et al. [11]. Chromatographic separations were executed on a HPLC system composed of a quaternary pump, an autosampler, and a diode array detector (DAD) (1260 Infinity, Agilent Technologies, Santa Clara, CA, USA). Desulfoglucosinolates were separated on a 4.6 mm × 250 mm, 5 μm, C18 reverse phase column (Luna, Phenomenex, Torrace, CA, USA). Separation of desulfoglucosinolates in the HPLC-DAD system was achieved using water (phase A) and acetonitrile (phase B) as mobile phases with a flow rate of 1.5 mL/min and a gradient of 0/100, 28/80, 30/100 (min/% phase A) with an injection volume of 20 µL. Desulfoglucosinolates were detected at 227 nm. Chromatographic data was processed with OpenLAB CDS ChemStation software (Agilent Technologies).

Mass spectra of compounds were obtained on a MS Finnigan LCQ Deca XP Max, Ion trap mass spectrometer coupled at the exit of the DAD and equipped with a Z-spray ESI source, and run by Xcalibur version 1.3 software (Thermo Finnigan-Surveyor, San José, CA, USA). Separations were conducted using the Phenomenex (Torrance, CA, USA) Synergi™ 4 µm Hydro-RP 80 Å (2 mm × 150 mm) with a C18 guard column. The gradient of the solvent system used was 0/99, 16/80, 18/10 (min/% phase A) and a flow rate of 350 µL/min from the DAD eluent was directed to the ESI interface using a flow-splitter. Nitrogen was used as desolvation gas at 275 °C and a flow rate of 60 L/h, and helium was used as damping gas. ESI was performed in the negative ion mode using the following conditions: sheath gas (N_2_), 60 arbitrary units; spray voltage, 5 kV; capillary temperature, 285 °C; capillary voltage, 48.5 V; and tube lens offset, 30 V.

Individual glucosinolates were identified on the basis of retention time, UV spectra, and their mass-to-charge (*m*/*z*) ratio as compared with authentic standards and previous literature data [2,7,11,25,60,61]. For the quantification of glucosinolates, a standard curve of desulfoglucoraphanin was prepared in the range of 0–700 μM. The concentration of total and individual glucosinolates was expressed as mmol of desulfoglucoraphanin equivalents per g of broccoli sprouts dry weight (DW).

#### 4.3.3. Analysis of Phenolic Compounds

##### Identification and Quantification of Phenolic Compounds by High-Performance Liquid Chromatography-Diode Array Detection (HPLC-DAD) and HPLC-Electrospray Ionization (ESI)-MS^n^

The identification and quantification of individual phenolic compounds were performed as described by Torres-Contreras et al. [52] with slight modifications according to Villarreal-García et al. [11]. Briefly, 10 µL of clarified methanolic or ethanolic extracts, previously filtered using 0.45 µm nylon membranes (VWR), were injected in the HPLC-DAD system (1260 Infinity, Agilent Technologies). Compounds were separated on a 4.6 mm × 250 mm, 5 µm particle size, C18 reverse phase column (Luna, Phenomenex). Mobile phases consisted of water (phase A) and methanol:water (60:40, *v*/*v*, phase B) both adjusted at pH 2.4 with orthophosphoric acid. The gradient solvent system was 0/100, 3/70, 8/50, 35/30, 40/20, 45/0, 50/0, and 60/100 (min/% phase A) at a constant flow rate of 0.8 mL/min. Phenolic compounds were detected at 280, 320 and 360 nm. Chromatographic data was processed with OpenLAB CDS ChemStation software (Agilent Technologies).

To obtain the mass spectra of compounds, the same HPLC solvent gradient was used for the HPLC-ESI-MS^n^ analyses, with mobile phases being adjusted to pH 2.4 with formic acid, and a flow rate of 200 µL/min. Nitrogen was used as desolvation gas at 275 °C and a flow rate of 60 L/h. Helium was used as damping gas. ESI was performed in the negative ion mode using the following conditions: sheath gas (N_2_), 60 arbitrary units; spray voltage, 1.5 kV; capillary temperature, 285 °C; capillary voltage, 45.7 V; and tube lens offset, 30 V.

Identification of individual phenolics was performed on the basis of retention time, UV spectra and their mass-to-charge ratio as compared with authentic standards and reported data [7,11,20,25,37,38,39,40,62]. To quantify phenolic compounds, standard curves of sinapic acid (0–100 ppm), ferulic acid (0–20 ppm), gallic acid (0–20 ppm) and 3-*O*-CQA (0–20 ppm) were prepared. Thus, the concentration of individual phenolic compounds was expressed as mg of sinapic acid, ferulic acid, gallic acid or 3-*O*-CQA equivalents per kg of broccoli sprouts DW, as appropriate. Similarly, the concentration of total phenolics (mg/kg DW) was determined as the sum of all individual phenolic compounds.

### 4.4. Statistical Analysis

Statistical analyses of chemical analyses were performed using three treatment repetitions. Data represent the mean values of samples and their standard error. Analyses of variance (ANOVA) were conducted using JMP software version 12.0 (SAS Institute Inc., Cary, NC, USA) and mean separations performed using the LSD test (*p* < 0.05).

## 5. Conclusions

Broccoli sprouts possess a high potential to manage against oxidative stress and, thus, act as strong anti-cancer as well as anti-degenerative ready-to-eat foods. Therefore, improving the phytochemical quality of these products is desirable. Results presented herein showed that UVA or UVB light exposure of broccoli sprouts can be used as a simple technology to enhance levels of specific secondary plant metabolites including glucosinolates and phenolic compounds.

For glucosinolates, UVB_H_ radiation and harvest after 24 h, resulted in the highest increase in both total and individual glucosinolates; followed by UVA_L_ radiation and harvest after 24 h. Both treatments led to the accumulation of aliphatic and indolyl glucosinolates; however, the former greatly favored the accumulation of 4-MGBS and GIB, whereas the latter favored overproduction of GIB and GBS.

As for phenolic content, a similar trend was observed, where UVB_H_ radiation and harvest after 24 h and UVA_L_ radiation and harvest after 2 h showed higher accumulation of individual phenolics. Both UV treatments increased 4-*O*-CQA; but UVA should be preferred if accumulation of sinapic acid, gallic acid and gallic acid derivatives is pursued while UVB treatment should be considered if phenolic glycosides are desired, as well as the UVB screening agent, sinapoyl malate.

Data herein presented suggests that both UVA and UVB radiations may interact with specific plant photoreceptors, triggering a signal transduction process that leads to the up-regulation of genes involved in the biosynthesis of UV-protective glucosinolates and phenolic compounds. In parallel, UV in broccoli sprouts may induce and interact with other signals including ethylene, NO and/or H_2_O_2_, which in turn activate genes related with the accumulation of secondary metabolites.

Thus, UV dose and harvesting time of broccoli sprouts could be exploited to differentially tailor glucosinolates and phenolic profiles and be a functional food for fresh consumption or a source of bioactive compounds with potential application in the nutraceutical foods, dietary supplements, pharmaceutical, cosmetic and skin care markets.

Further experiments should consider evaluating the effect of other types of radiation on the phytochemical content of broccoli sprouts. For instance, it has been reported that gamma radiation affect the phenolic content of plants such as *Phaseolus vulgaris* [63], *Moringa oleifera* [64] and *Aloysia citrodora* Paláu [65]. Moreover, further research should evaluate the effect of radiation on the isomerization of phytochemicals identified in broccoli sprouts, especially cinnamic acids because they are highly susceptible to isomerization [66], and their bioactivity can be modified.

## Figures and Tables

**Table 1:** Identification of individual desulfoglucosinolates (dsg) in broccoli sprouts. Identification was obtained by HPLC-DAD and HPLC-ESI-MS^n^.

Peak Number (Retention Time, min)	λ_max_ (nm)	Identification	[M − H]^−^ (*m*/*z*)	MS^2^ (*m*/*z*) ^a^
1 (5.3)	222	Glucoiberin-dsg	342	**179**, 131
2 (5.8)	224	Progoitrin-dsg	308	**145**, 129, 79
3 (6.6)	222	Glucoraphanin-dsg	356	**193**
4 (13.6)	221, 266	4-hydroxy-glucobrassicin-dsg	383	**221**, 203, 153
5 (17.9)	210	Glucoerucin-dsg	340	**177**, 160, 129, 113
6 (20.6)	220, 280	Glucobrassicin-dsg	367	**204**, 187, 155, 129
7 (24.3)	220, 268	4-methoxy-glucobrassicin-dsg	397	**234**, 204, 154, 139

^a^ Major fragment ions are highlighted in bold.

**Table 2:** Concentration of total and individual glucosinolates in broccoli sprouts treated with UVA or UVB light.

	Dose ^4^	Solvent	Time of Harvest after Treatment ^5^	Glucosinolate Concentration (mmol/kg DW) ^1,2,3^															
				GIB		PRO		GRA		4-HGBS		GER		GBS		4-MGBS		Total	
Control		M	2 h	4.63 ± 0.2	ij	0.31 ± 0.03	hi	18.61 ± 0.4	h	15.52 ± 0.75	d	9.03 ± 0.41	efgh	7.79 ± 0.23	g	6.56 ± 0.54	jk	62.45 ± 1.44	ij
		E		5.09 ± 0.08	ghi	0.39 ± 0.06	fgh	22.1 ± 0.57	efg	12.16 ± 0.71	ghi	6.14 ± 0.68	klm	7.33 ± 0.2	g	5.69 ± 0.28	l	58.9 ± 1.71	jk
		M	24 h	4.71 ± 0.15	hij	0.38 ± 0.02	fgh	18.47 ± 0.3	h	15.49 ± 0.73	de	10.91 ± 0.14	cd	9.26 ± 0.08	ef	7.35 ± 0.08	ghi	66.56 ± 0.87	hi
		E		5.32 ± 0.02	fghi	0.31 ± 0.04	hi	21.67 ± 0.43	fg	14.85 ± 0.43	defg	8.67 ± 0.43	fghi	9.51 ± 0.28	def	7.42 ± 0.19	ghi	67.76 ± 0.77	ghi
UVA	UVA_L_	M	2 h	3.74 ± 0.03	k	0.31 ± 0.01	hi	15.42 ± 0.08	i	12.71 ± 0.94	efghi	8.05 ± 0.17	ghij	8.02 ± 0.24	fg	7.09 ± 0.24	ij	55.35 ± 1.52	jk
		E		4.05 ± 0.03	jk	0.32 ± 0.02	hi	18.18 ± 0.5	h	10.09 ± 0.6	i	5.77 ± 0.2	m	7.38 ± 0.08	fg	6.79 ± 0.21	ij	52.59 ± 1.12	k
		M	24 h	7.06 ± 0.34	d	0.64 ± 0.07	ab	26.54 ± 1.2	bc	19.99 ± 1.2	a	12.19 ± 0.46	bc	14.77 ± 0.46	a	9.05 ± 0.23	e	90.24 ± 3.38	c
		E		7.98 ± 0.15	bc	0.58 ± 0.01	abcd	31.8 ± 0.76	a	19.19 ± 1.65	ab	9.66 ± 0.85	def	14.52 ± 0.35	a	9.14 ± 0.29	e	92.89 ± 4.07	bc
	UVA_H_	M	2 h	6.54 ± 0.24	de	0.34 ± 0.01	gh	23.79 ± 1.2	def	18.72 ± 1.03	abc	11.93 ± 0.2	bc	12.52 ± 0.09	b	7.87 ± 0.13	fg	81.71 ± 1.38	d
		E		7.21 ± 0.12	cd	0.42 ± 0.02	efgh	28.16 ± 0.35	b	15.98 ± 1.1	cd	7.54 ± 0.68	ijk	11.25 ± 0.43	bc	7.37 ± 0.31	ghi	77.94 ± 2.46	de
		M	24 h	5.39 ± 0.09	fgh	0.7 ± 0.04	a	19.66 ± 0.47	gh	16.98 ± 0.05	bcd	10.93 ± 0.18	bcd	12.46 ± 0.07	b	8.53 ± 0.11	ef	74.66 ± 0.39	defg
		E		5.39 ± 0.35	fgh	0.61 ± 0.03	abc	21.39 ± 1.58	fg	14.2 ± 0.47	defgh	7.62 ± 0.22	hij	11.63 ± 0.06	bc	7.83 ± 0.11	fgh	68.65 ± 2.3	fghi
UVB	UVB_L_	M	2 h	6.74 ± 0.57	d	0.45 ± 0.07	defg	24.76 ± 1.52	cde	18.93 ± 1.17	ab	11.6 ± 0.55	bc	12.56 ± 0.19	b	6.7 ± 0.27	ijk	81.75 ± 3.97	d
		E		7.07 ± 0.05	d	0.51 ± 0.06	cde	28.5 ± 0.32	b	14.96 ± 1.56	def	7.46 ± 0.54	ijk	11.06 ± 0.32	bcd	6.03 ± 0.24	kl	75.59 ± 2.69	def
		M	24 h	5.96 ± 0.43	ef	0.21 ± 0.04	i	24.87 ± 1.95	cd	14.8 ± 1.84	defg	9.41 ± 1.08	efg	12.34 ± 0.69	b	11.47 ± 0.48	c	79.05 ± 6.39	d
		E		5.52 ± 0.06	fg	0.31 ± 0.06	hi	23.43 ± 0.43	def	10.5 ± 1.01	i	6.04 ± 0.35	lm	10.41 ± 0.35	cde	9.99 ± 0.22	d	66.19 ± 1.71	hi
	UVB_H_	M	2 h	6.59 ± 0.32	de	0.52 ± 0.03	bcde	24.75 ± 0.32	cde	14.92 ± 0.9	defg	10.18 ± 0.27	de	11.38 ± 0.26	bc	7.1 ± 0.1	hij	75.44 ± 1.93	def
		E		7.12 ± 0.02	d	0.47 ± 0.03	def	27.86 ± 0.23	b	11.66 ± 0.33	hi	7.24 ± 0.21	jkl	10.14 ± 0.07	cde	6.83 ± 0.14	ij	71.31 ± 0.38	efgh
		M	24 h	8.9 ± 0.48	a	0.62 ± 0.05	abc	31.86 ± 1.72	a	15.9 ± 0.91	d	15.2 ± 0.61	a	16.45 ± 1.56	a	19.86 ± 0.25	a	108.79 ± 2.07	a
		E		8.72 ± 0.2	ab	0.51 ± 0.05	bcdef	33.33 ± 0.84	a	12.34 ± 0.89	fghi	12.52 ± 1.02	b	15.55 ± 3.01	a	18.2 ± 0.36	b	101.17 ± 2.4	ab

^1^ Concentrations are reported as desulfoglucoraphanin equivalents. All compounds were quantified at 227 nm; ^2^ Values represent the mean of three replicates ± standard error of the mean; ^3^ Different letters in the same column indicate statistical difference in the concentration of each compound between treatments using the LSD test (*p* < 0.05); ^4^ UV doses were 3.16, 4.05, 2.28 or 3.34 W/m^2^ for 120 min for treatments UVA_L_, UVA_H_, UVB_L_ and UVB_H_, respectively; ^5^ All UVA or UVB treatments occurred at the 7th day after sowing. Harvest of treated sprouts was performed 2 h or 24 h after the UV treatment. For control sprouts, harvest occurred at the 7th day + 2 h or 24 h after sowing, without any treatment. Abbreviations: 70% Methanol (M); 70% Ethanol (E); Glucoiberin (GIB); Progoitrin (PRO); Glucoraphanin (GRA); 4-hydroxy-glucobrassicin (4-HGBS); Glucoerucin (GER); Glucobrassicin (GBS); 4-methoxy-glucobrassicin (4-MGBS).

**Table 3:** Identification of individual phenolic compounds in broccoli sprouts. Identification was obtained by HPLC-DAD and HPLC-ESI-MS^n^.

Peak Number (Retention Time, min)	λ_max_ (nm)	Identification	[M − H]^−^ (*m*/*z*)	MS^2^ (*m*/*z*) ^a^
1 (4.2)	262	Gallic acid hexoside I	331	162, **125**
2 (6.9)	210, 300	Gallotannic acid	1700	1530, **1378**, 1225, 1091
3 (10.7)	272	*p*-Hydroxybenzoic acid	137	122, **111**, 107
4 (11.8)	218, 280	Gallic acid hexoside II	331	**162**, 125
5 (12.2)	218 sh, 326 sh	4-*O*-Caffeoylquinic acid	353	**191**, 179, **173**
6 (12.7)	220, 268	Digalloyl hexoside	483	337, **169**
7 (13.6)	222, 265, 330	3-*O*-Hexoside kaempferol	447	**285**
8 (14.6)	220, 268	Gallic acid derivative	-	-
9 (15.3)	240 sh, 328	1-*O*-Sinapoyl-β-d-glucose	385	**223**, 205, **173**, 145
10 (16.2)	240 sh, 330	Sinapoyl malate	339	**205.6**, **173**, 147, 132
11 (17.2)	228, 330	1,2-Diferuloylgentiobiose	693	499, **175**
12 (22.5)	220, 268	5-Sinapoylquinic acid	397	**222**, 191
13 (27.1)	235, 324	Sinapic acid	223	**179**, **163**, 135, 119
14 (29.3)	221, 290	Gallic acid	169	167, 141, **137**, 125, 81
15 (36.2)	238 sh, 270, 330	Kaempferol 3-*O*-sinapoyl-sophoroside 7-*O*-glucoside	977	771, **609**, 429, **285**
16 (37.6)	240 sh, 268, 332	1,2-Disinapoylgentiobiose	753	529, **223**
17 (39.9)	240 sh, 330	1-Sinapoyl-2′-ferulolylgentiobiose	723	449, **223**
18 (42.4)	240 sh, 328	1,2,2′-Trisinapoylgentiobiose ^b^	959	735, **223**
19 (43.2)	240 sh, 331	1,2-Disinapoyl-1′-ferulolylgentiobiose	929	705, **223**
20 (43.9)	220, 238, 328	1,2-Disinapoyl-2′-ferulolylgentiobiose	929	705, **223**
21 (46.6)	242, 326	1-Sinapoyl-2,2′-diferuloylgentiobiose	899	705, **223**
22 (51.2)	238 sh, 330	1,2,2′-Trisinapoylgentiobiose ^b^	959	735, **223**

Abbreviations: Shoulder (sh). ^a^ Major fragment ions are highlighted in bold; ^b^ Isomeric compounds.

**Table 4:** Concentration of total and individual phenolic compounds in broccoli sprouts treated with UVA or UVB light.

	**Dose ^5^**	**Solvent**	**Time of Harvest after Treatment ^6^**	**Phenolic Concentration (mg/kg DW) ^1,2,3,4^**															
				**GAH I**		**GTA**		***p*-HBA**		**GAH II**		**4-*O*-CQA**		**diGH**		**3-*O*-H-K**		**GAD**	
Control		M	2 h	411.2 ± 8.5	c	171.3 ± 9.7	d	428.7 ± 18.8	a	423.5 ± 5.4	a	138 ± 8	bcde	202.6 ± 14.2	bcde	195.6 ± 1.8	abcd	67.7 ± 5.2	ef
		E		290.9 ± 0.7	g	98.5 ± 22.8	e	260.6 ± 21.3	fghi	322.1 ± 6.2	bc	86.2 ± 14.8	f	160.1 ± 10.3	h	136.3 ± 11.3	hi	74.8 ± 0.7	de
		M	24 h	439.6 ± 10.7	b	195.4 ± 3.3	bcd	424.4 ± 13.6	a	430.9 ± 9.5	a	122.9 ± 21.6	bcdef	221.2 ± 2.8	bc	200.9 ± 4.6	abc	70.6 ± 2.1	ef
		E		324.7 ± 4.8	f	61.5 ± 9.3	ef	312.2 ± 10.4	cde	337.5 ± 1.7	bc	90.6 ± 1.1	ef	173.4 ± 5.5	fgh	145.9 ± 30.4	gh	75.9 ± 5.3	cde
UVA	UVA_L_	M	2 h	469.2 ± 4.3	a	197.2 ± 8.6	bcd	354 ± 13.6	bcd	267 ± 7.6	efg	137.7 ± 16.5	bcde	155.7 ± 4.6	h	211.6 ± 12.7	ab	85.2 ± 0.7	bcd
		E		357.1 ± 4.7	de	91.7 ± 29	e	251 ± 15.6	ghi	225 ± 4.4	hij	196.2 ± 2.2	a	120 ± 7	i	219.9 ± 3	a	100 ± 2.7	a
		M	24 h	339.2 ± 10.7	ef	228.1 ± 18.5	b	273.9 ± 21.3	efghi	250.2 ± 9.2	fgh	134.6 ± 33.3	bcdef	269.6 ± 13.7	a	162.7 ± 5.4	efgh	65.7 ± 2	ef
		E		260.5 ± 4.2	hi	58.4 ± 13	ef	221.6 ± 4.6	i	199.5 ± 4.4	jk	169.7 ± 3.9	abc	195 ± 24.1	cdef	184.6 ± 2.1	bcdef	60.6 ± 0.2	f
	UVA_H_	M	2 h	346 ± 8.4	ef	173.9 ± 10.2	d	390.5 ± 27.9	ab	269.6 ± 5.1	efg	106.6 ± 17	def	220.3 ± 5.4	bc	158.5 ± 7.5	fgh	71.1 ± 1.8	ef
		E		261.1 ± 6.2	hi	66.9 ± 3.3	ef	246.6 ± 9.3	hi	221.6 ± 0.7	hij	134.4 ± 14.2	bcdef	156.9 ± 3.5	h	160.3 ± 12.3	fgh	71.2 ± 4	ef
		M	24 h	373.1 ± 20.4	d	178.8 ± 16.2	d	357.8 ± 25.8	bc	272.5 ± 7.6	def	161 ± 25.3	abc	222.5 ± 12.1	b	192.1 ± 4.6	abcde	67.5 ± 2	ef
		E		291.6 ± 13	g	99.8 ± 28.2	e	284.9 ± 13.1	efgh	223 ± 10.9	hij	169.9 ± 7.9	ab	165.9 ± 7.4	gh	198.9 ± 4.6	abc	73.6 ± 7.5	def
UVB	UVB_L_	M	2 h	410.6 ± 3.3	c	225.4 ± 6	bc	305.4 ± 20.8	ef	291.5 ± 5.5	de	152.9 ± 35.4	abcd	224.6 ± 4.1	b	211.3 ± 1.7	ab	94.1 ± 3.4	ab
		E		328.7 ± 4.2	f	78.6 ± 10.6	ef	238.5 ± 7.8	hi	242.3 ± 5	ghi	164.9 ± 3	abc	188.8 ± 6.6	defg	212.1 ± 4.8	ab	93.4 ± 7.8	ab
		M	24 h	400.3 ± 3.9	c	193.7 ± 10.4	bcd	417.5 ± 12.1	a	350.2 ± 37	b	195.5 ± 7.3	a	187.2 ± 2.6	efg	188.3 ± 2	bcdef	85.1 ± 0.4	bcd
		E		293.4 ± 4.5	g	102.5 ± 19.5	e	297.3 ± 10.2	efg	243.8 ± 1.1	fghi	165.5 ± 5.9	abc	130.2 ± 3.2	i	186 ± 2.4	bcdef	85.7 ± 9.4	bcd
	UVB_H_	M	2 h	370.1 ± 1.8	d	185 ± 3.3	cd	399 ± 17.3	ab	218.7 ± 4.3	ij	116.9 ± 7.3	cdef	212.4 ± 9.8	bcf	170.1 ± 6.7	cdefg	96.6 ± 2.1	ab
		E		293.6 ± 1.1	g	47.2 ± 5	f	307.9 ± 3.3	de	188.1 ± 2.7	k	160.7 ± 6.9	abc	131.5 ± 5.2	i	162.1 ± 21.5	efgh	93.2 ± 6.8	ab
		M	24 h	335.4 ± 6.1	ef	288.6 ± 6.2	a	313.4 ± 21	cde	300.5 ± 6.6	cd	136.9 ± 28.2	bcde	218.7 ± 7.5	bc	166.8 ± 9.4	defgh	88.3 ± 1.1	abc
		E		235.1 ± 0	i	38.4 ± 12.2	f	289.2 ± 19.7	efgh	221.9 ± 6.4	hij	119.7 ± 3.5	bcdef	160.6 ± 1.6	h	102.3 ± 19.9	i	75.8 ± 1.5	cdef
	**Dose ^5^**	**Solvent**	**Time of Harvest after Treatment ^6^**	**Phenolic Concentration (mg/kg DW) ^1,2,3,4^**															
				**1-*O*-S-β-d-g**		**Sinapoyl Malate**		**1,2-diFG**		**5-SQA**		**Sinapic Acid**		**Gallic Acid**		**K-3-*O*-S-so-7-*O*-g**		**1,2-diSG**	
Control		M	2 h	241.5 ± 4.9	ab	1600.8 ± 25	efg	152.2 ± 5.7	abcd	81.5 ± 5.5	gh	307.5 ± 9.2	b	168.8 ± 11.3	abc	276.8 ± 2.6	cde	178.3 ± 3.2	abcd
		E		181.9 ± 0.9	fg	1471.5 ± 5	i	121.7 ± 2.4	def	48.1 ± 4.1	k	245.6 ± 1.8	cd	136.2 ± 5.4	fgh	261.9 ± 26.1	de	139.8 ± 1.8	g
		M	24 h	251.6 ± 4.9	a	1451.8 ± 10.7	i	152.2 ± 8.7	abcd	89.6 ± 2.7	efg	300.8 ± 3.1	b	163.8 ± 5.1	bcde	359.2 ± 37.5	abc	194.4 ± 3.8	a
		E		205.2 ± 4	cd	1320.6 ± 27.3	j	121.3 ± 22.2	def	64.9 ± 1.2	j	245.3 ± 5.4	cd	143.4 ± 15.5	defgh	296.5 ± 58.3	bcde	146.5 ± 15.4	fg
UVA	UVA_L_	M	2 h	235.4 ± 3.4	b	1612.6 ± 20.4	def	175.7 ± 3.7	a	80.9 ± 1	gh	328.9 ± 11.5	a	167.2 ± 6	abcd	356.9 ± 6.7	abc	190.8 ± 2.7	ab
		E		202.8 ± 4.9	cde	1565.8 ± 11.3	fgh	106.3 ± 17.3	fg	65.5 ± 1.9	j	293.7 ± 4.4	b	164.6 ± 9.7	bcde	338 ± 22.5	abcd	158.9 ± 2.3	defg
		M	24 h	169.6 ± 5.1	hij	1718.7 ± 63.8	abc	118 ± 16.1	efg	105.5 ± 6.4	cd	150.6 ± 5.1	i	156.4 ± 6.3	bcdefg	322.9 ± 45.7	abcde	166.1 ± 12.5	cdef
		E		136.1 ± 0.5	k	1596.3 ± 37.1	efg	129.6 ± 1.2	cdef	66.6 ± 1.6	ij	119.9 ± 2.3	j	135.6 ± 3.3	fghi	397.5 ± 8.9	a	145.2 ± 3.9	fg
	UVA_H_	M	2 h	178.9 ± 2.6	gh	1657.1 ± 46.5	bcde	144.6 ± 2.5	abcde	93.1 ± 4.6	ef	218.2 ± 6	ef	191.8 ± 11.1	a	253.5 ± 4.3	ef	163.8 ± 5.3	cdef
		E		148.6 ± 3.7	k	1626.9 ± 29.8	def	119.3 ± 3.4	defg	59.2 ± 1.6	j	186.9 ± 1.4	h	145.8 ± 10	cdefg	177 ± 4.9	f	111 ± 1.5	h
		M	24 h	199.6 ± 5.6	de	1632.9 ± 24.6	cdef	128.7 ± 25.2	cdef	98.8 ± 6.6	de	207.4 ± 13.5	fg	179.5 ± 1.8	ab	330.1 ± 16	abcde	192.7 ± 5.4	a
		E		171.3 ± 3.4	ghi	1583.5 ± 48.2	efg	60.8 ± 8.2	h	76.2 ± 1.5	hi	177.5 ± 10.3	h	160.1 ± 7.6	bcdef	333 ± 43.2	abcde	167 ± 4.9	cdef
UVB	UVB_L_	M	2 h	230.8 ± 2.2	b	1784.1 ± 18.7	a	170.3 ± 2.5	ab	81.3 ± 1.8	gh	257.1 ± 1.4	c	152.5 ± 2.7	cdefg	308.7 ± 12.7	abcde	189.5 ± 11.9	ab
		E		199.7 ± 8.3	de	1669.2 ± 45.5	bcde	136.5 ± 2.6	bcdef	60.2 ± 1.2	j	232.7 ± 5.2	de	133.5 ± 2.3	ghi	250.2 ± 28	ef	146.3 ± 13.4	fg
		M	24 h	210.9 ± 0.5	c	1622.8 ± 36.5	def	151 ± 7	abcde	111.4 ± 3.6	c	222.3 ± 3.2	ef	155.5 ± 4.5	bcdefg	282.2 ± 38.4	bcde	170.1 ± 7.8	bcde
		E		162.9 ± 3.3	j	1491.9 ± 13.7	hi	123.4 ± 1	def	91.8 ± 1.8	ef	177.4 ± 10	h	140.5 ± 3.8	efgh	298.1 ± 31.1	bcde	145.2 ± 1.7	fg
	UVB_H_	M	2 h	192.4 ± 1.6	ef	1740.9 ± 6.3	ab	158 ± 2.5	abc	86.4 ± 1.9	fg	211 ± 3.8	fg	120.9 ± 9	hi	305.2 ± 20.7	abcde	177.9 ± 11.8	abcd
		E		160 ± 2	j	1636.7 ± 31.2	cdef	86.9 ± 16.6	gh	59.5 ± 2.4	j	196.1 ± 10	gh	110.7 ± 11.3	i	345 ± 14.6	abcd	153 ± 1	efg
		M	24 h	171.4 ± 2.6	ghi	1700.6 ± 25.1	abcd	137.8 ± 20.8	bcdef	197.6 ± 5.2	a	144.5 ± 3.6	i	164.7 ± 6.2	bcde	323.3 ± 43.2	abcde	181.4 ± 9.8	abc
		E		121.1 ± 2.2	l	1502 ± 8.9	ghi	124.3 ± 2.2	cdefg	146.7 ± 3.9	b	90.4 ± 4.7	k	130.6 ± 26.5	ghi	371.5 ± 26.6	ab	154.6 ± 1.9	defg
	**Dose ^5^**	**Solvent**	**Time of Harvest after Treatment ^6^**	**Phenolic Concentration (mg/kg DW) ^1,2,3,4^**															
				**1-S-2-FG**		**1,2,2-triSG ^7^**		**1,2-diS-1-FG**		**1,2-diS-2-FG**		**1-S-2,2-diFG**		**1,2,2-triSG ^7^**		**Total**			
Control		M	2 h	1505.7 ± 24.7	hij	4394.1 ± 58.8	cde	1107.9 ± 38.5	a	162.6 ± 0.9	bc	89.5 ± 1	h	119.9 ± 2.2	ef	12,425.7 ± 180.6		bc	
		E		1374.7 ± 6.7	k	4069.6 ± 8.2	gh	1010.4 ± 14.8	cd	127.2 ± 1	k	55.9 ± 3.2	i	85.7 ± 1.3	i	10,759.8 ± 37.1		ghi	
		M	24 h	1729.6 ± 19.1	abcd	4793.6 ± 46.8	a	1134.9 ± 17.2	a	166.9 ± 2.2	b	100.2 ± 3.1	gh	139.2 ± 1.1	abc	13,133.6 ± 176.9		a	
		E		1608.4 ± 24.9	efg	4561.7 ± 87	bcd	1089.2 ± 19.8	ab	134.6 ± 3.1	hij	68.8 ± 2.2	i	95.3 ± 12	hi	11,623.2 ± 264.3		def	
UVA	UVA_L_	M	2 h	1737.7 ± 18.6	abcd	4261.3 ± 23.8	efg	840.7 ± 9.7	gh	162.3 ± 0.9	bcd	144.3 ± 2.4	a	126.3 ± 1.4	de	12,298.6 ± 123.3		c	
		E		1703.9 ± 17.6	bcd	4252 ± 60.4	efg	794.4 ± 11.3	h	137.2 ± 2.9	hi	111.2 ± 4.2	efg	98.4 ± 1.4	h	11,553.6 ± 130.3		ef	
		M	24 h	1735 ± 69	abcd	4240.1 ± 169.4	efg	892.1 ± 42.5	efg	163.8 ± 5.8	bc	132.9 ± 4.3	abc	130 ± 4.5	cd	11,925.8 ± 430.1		cde	
		E		1645.5 ± 44.5	defg	4126 ± 63.1	fgh	835.2 ± 11.1	gh	130.5 ± 0	ijk	100.5 ± 7.1	fgh	103.1 ± 2.6	gh	11,017.6 ± 204.2		fgh	
	UVA_H_	M	2 h	1495.3 ± 48.6	ij	3807.9 ± 123.6	i	953.7 ± 22.6	de	145.8 ± 3.2	fg	128.6 ± 4.2	bc	115.2 ± 1.9	f	11,284 ± 297.4		fg	
		E		1450.5 ± 30.3	jk	3735.8 ± 76.8	i	919.9 ± 23.1	ef	114.4 ± 3.5	m	98.6 ± 6.8	gh	87.5 ± 2	i	10,300.4 ± 141.2		i	
		M	24 h	1784.4 ± 39.4	ab	4390 ± 102.5	cde	953.1 ± 15.9	de	155.3 ± 2.4	de	139.2 ± 1.9	ab	133.9 ± 2.2	cd	12,351 ± 247.6		c	
		E		1759.8 ± 45.5	abc	4360.5 ± 118.5	def	950.1 ± 20.7	de	128.7 ± 2.9	jk	96.4 ± 2.6	h	111.4 ± 2.6	fg	11,643.7 ± 313.9		def	
UVB	UVB_L_	M	2 h	1809.8 ± 23.6	a	4645.6 ± 75.9	ab	1126.3 ± 14.5	a	165.8 ± 2.7	b	136.4 ± 7.6	abc	136.6 ± 1.3	bc	13,110.7 ± 252.6		a	
		E		1760.6 ± 32.2	abc	4588.5 ± 100.7	abc	1099.6 ± 35.1	a	138.4 ± 1.7	hi	115.1 ± 1	def	112 ± 2.6	fg	12,189.7 ± 281.9		cd	
		M	24 h	1675.6 ± 16.6	cde	4350 ± 57.7	def	1027.5 ± 34.3	bc	158.6 ± 1.4	cd	123.2 ± 5.1	cde	145.7 ± 2.5	ab	12,424.7 ± 116.4		bc	
		E		1587.6 ± 8.9	fgh	4091.1 ± 23.1	gh	951.2 ± 24.8	de	125.1 ± 1.2	kl	99.7 ± 6.5	gh	114.4 ± 2.8	f	11,104.7 ± 54.3		fgh	
	UVB_H_	M	2 h	1651 ± 22.2	def	3925 ± 16.2	hi	933.7 ± 17.3	ef	149.7 ± 0.7	ef	128 ± 6.4	bcd	114.5 ± 1.5	f	11,663.4 ± 95.4		def	
		E		1553 ± 19.1	ghi	3814.4 ± 71.8	i	873.8 ± 13.8	fg	119.1 ± 2.3	lm	91.5 ± 5.2	h	86 ± 0.4	i	10,669.9 ± 119.5		hi	
		M	24 h	1812.8 ± 14.2	a	4717.6 ± 43.9	ab	1087.7 ± 22	ab	176 ± 0.7	a	140.3 ± 6.1	ab	147.2 ± 1.7	a	12,951.5 ± 97.1		ab	
		E		1654 ± 5.3	def	4351 ± 7.2	cdef	964.2 ± 3.8	cde	139.8 ± 0.4	gh	102 ± 7.5	fgh	114 ± 1.9	fg	11,209 ± 36.7		fgh	

^1^ Concentrations are reported as gallic acid equivalents for GAH I, GTA, *p*-HBA, GAH II, diGH, GAD and gallic acid; as 3-*O-*CQA equivalents for 4-*O*-CQA; as ferulic acid equivalents for 1,2-diFG; and as sinapic acid equivalents for 3-*O-*H-K, 1-*O*-S-β-d-g, sinapoyl malate, 5-SQA, sinapic acid, K-3-*O*-S-so-7-*O*-g, 1,2-diSG, 1-S-2-FG, 1,2,2-triSG, 1,2-diS-1-FG, 1,2-diS-2-FG and 1-S-2-diFG; ^2^ Compounds quantified at 280 nm (GAH I, GTA, *p*-HBA, GAH II, diGH, GAD and gallic acid) and at 320 nm (4-*O*-CQA, 1,2-diFG, 3-*O*-H-K, 1-*O*-S-β-d-g, sinapoyl malate, 5-SQA, sinapic acid, K-3-*O*-S-so-7-*O*-g, 1,2-diSG, 1-S-2-FG, 1,2,2-triSG, 1,2-diS-1-FG, 1,2-diS-2-FG and 1-S-2-diFG); ^3^ Values represent the mean of three replicates ± standard error of the mean; ^4^ Different letters in the same column indicate statistical difference in the concentration of the compound between treatments using the LSD test (*p* < 0.05); ^5^ UV doses were 3.16, 4.05, 2.28 or 3.34 W/m^2^ for 120 min for treatments UVA_L_, UVA_H_, UVB_L_ and UVB_H_, respectively; ^6^ All UVA or UVB treatments occurred at the 7th day after sowing. Harvest of treated sprouts was performed 2 h or 24 h after the UV treatment. For control sprouts, harvest occurred at the 7th day + 2 h or 24 h after sowing, without any treatment. ^7^ Isomeric compounds. Abbreviations: 70% Methanol (M); 70% Ethanol (E); gallic acid hexoside I (GAH I); gallotannic acid (GTA); *p*-hydroxybenzoic acid (*p*-HBA); gallic acid hexoside II (GAH II); 4-*O*-caffeoylquinic acid (4-*O*-CQA); digalloyl hexoside (diGH); 3-*O*-hexoside kaempferol (3-*O*-H-K); gallic acid derivative (GAD); 1-O-sinapoyl-β-d-glucose (1-*O*-S-β-d-g); 1,2-diferulolylgentiobiose (1,2-diFG); 5-sinapoylquinic acid (5-SQA); kaempferol 3-*O*-sinapoyl-sophoroside 7-*O*-glucoside (K-3-*O*-S-so-7-*O*-g); 1,2-disinapoylgentiobiose (1,2-diSG); 1-sinapoyl-2′-ferulolylgentiobiose (1-S-2-FG); 1,2,2′-trisinapoylgentiobiose (1,2,2-triSG); 1,2-disinapoyl-1′-ferulolylgentiobiose (1,2-diS-1-FG); 1,2-disinapoyl-2-ferulolylgentiobiose (1,2-diS-2-FG); 1-sinapoyl-2,2′-diferulolylgentiobiose (1-S-2-diFG).
